# Limited response of NK92 cells to *Plasmodium falciparum*-infected erythrocytes

**DOI:** 10.1186/1475-2875-10-311

**Published:** 2011-10-21

**Authors:** Elisandra Grangeiro de Carvalho, Evelyn Böttger, Van Tong Hoang, Peter G Kremsner, Jürgen FJ Kun

**Affiliations:** 1 Institute for Tropical Medicine, Tübingen University, Wilhelmstrasse, 27; 72074 Tübingen; Germany

## Abstract

**Background:**

Mechanisms by which anti-malarial immune responses occur are still not fully clear. Natural killer (NK) cells are thought to play a pivotal role in innate responses against *Plasmodium falciparum*. In this study, the suitability of NK92 cells as models for the NK mechanisms involved in the immune response against malaria was investigated.

**Methods:**

NK92 cells were assessed for several signs of activation and cytotoxicity due to contact to parasites and were as well examined by oligonucleotide microarrays for an insight on the impact *P. falciparum*-infected erythrocytes have on their transcriptome. To address the parasite side of such interaction, growth inhibition assays were performed including non-NK cells as controls.

**Results:**

By performing microarrays with NK92 cells, the impact of parasites on a transcriptional level was observed. The findings show that, although not evidently activated by iRBCs, NK92 cells show transcriptional signs of priming and proliferation. In addition, decreased parasitaemia was observed due to co-incubation with NK92 cells. However, such effect might not be NK-specific since irrelevant cells also affected parasite growth *in vitro*.

**Conclusions:**

Although NK92 cells are here shown to behave as poor models for the NK immune response against parasites, the results obtained in this study may be of use for future investigations regarding host-parasites interactions in malaria.

## Background

More than any other disease restricted to tropical areas, malaria has a widespread impact and is considered one of the main public health problems in the world. The disease causes thousands of deaths annually and its burden continues to grow especially in areas of poverty.

The human immune system fails to completely eliminate malarial infections and the reason for this is still not known. Nevertheless, it is clear that immunity to malaria involves the innate and adaptive arms of the immune system, engaging macrophages, dendritic cells, γδT cells, Natural Killer T (NKT) and NK cells to participate in the response developed by the host against parasites [1,2]. Natural killer lymphocytes are thought to play an important role in combating infections. Without requiring clonal expansion ("naturally") and balanced by a repertoire of activating and inhibitory receptors, these cells are promptly triggered to develop their biological functions: cytotoxicity, cytokine and chemokine secretion and, therefore, co-stimulation of other cells of the immune system [3].

Experimental evidence suggested that NK cells are one of the first cells to sense a malarial infection and produce type 2 interferon [4-6]. Interferon-γ is described to be important for limiting parasitaemia in early infections. It presumably inhibits parasite development in hepatocytes and activates macrophages to promote phagocytose of intra-erythrocytic parasites and merozoites. Indeed, the need of accessory cells for complete NK activation via cross talk with dendritic cells and monocytes was already reported [7-9]. Moreover, killer cells derived from patients with malaria as well as from donors with no prior exposure to the disease were described to be cytotoxic to and lyse *Plasmodium*-infected erythrocytes (iRBCs) [10,11].

The immune response in malaria has been extensively investigated over the years. However, further studies are still required for a clear knowledge of the many unresolved issues regarding the *in vivo *functions of NK cells in malaria. NK cell lines are potential resources frequently adopted in studies aiming to investigate pathological mechanisms, particularly in diseases where primary material is of difficult access. A valuable use of these cells includes attempts to mimic the processes by which fresh NK cells recognize non-self, stress induced-self and missing-self molecules that trigger their activation and further response to infections.

The well-characterized NK92 cell line was already shown to directly interact with red blood cells infected with *P. falciparum *[4,5]. With the notion that once a model is appropriate it can be useful for understanding the behaviour of a system, the NK cell and the *Plasmodium *side of such host-parasite interaction was investigated to examine whether NK92 cells can be used as models for the mechanisms involved in the NK fight against malaria.

## Methods

### Cells

The NK92 cell line was purchased from the German Resource Centre for Biological Material (DSMZ, Braunschweig, Germany) and kept in culture at 0.2-0.6 × 10^6 ^cells/ml in alpha-MEM (Sigma-Aldrich) supplemented with FBS (12,5%; Sigma-Aldrich), horse serum (12,5%; Sigma-Aldrich), L-glutamine (2 mM; Sigma-Aldrich), penicillin-streptomycin (10 ml/L; Invitrogen) and recombinant human interleukin-2 (rIL-2; 10 ng/ml; Invitrogen).

Jurkat cells were obtained from the German Resource Centre for Biological Material (DSMZ; Braunschweig, Germany). Cells were kept in culture at 0.2-0.6 × 10^6 ^cells/ml in RPMI 1640 (Sigma-Aldrich) supplemented with FBS (10%; Sigma-Aldrich), L-glutamine (2 mM; Sigma-Aldrich) and penicillin-streptomycin (10 ml/L; Invitrogen).

HeLa cells were purchased from the German Resource Centre for Biological Material (DSMZ; Braunschweig, Germany). Cells were grown to maximum 70%/80% confluency in DMEM (Sigma-Aldrich) supplemented with FBS (10%; Sigma-Aldrich), L-glutamine (2 mM; Sigma-Aldrich) and penicillin-streptomycin (10 ml/L; Invitrogen).

C32 cells were obtained from American Type Culture Collection (ATCC; Rockville, MA, USA). Cells were grown to maximum 70%/80% confluency in DMEM (Sigma-Aldrich) supplemented with FBS (10%; Sigma-Aldrich), L-Glutamine (2 mM; Sigma-Aldrich), Gentamycin (50 μg/ml; Invitrogen) and MEM non-essential amino acid solution (1%; Sigma-Aldrich).

Mycoplasma free cells of maximum 12^th ^passage were utilized for all the experiments described in this study.

### *Plasmodium falciparum *culture

The laboratory strains *P. falciparum *3D7, FCR3-CSA, FCR3-CD36 and Dd2 were maintained in continuous culture as described elsewhere [12] and frequently tested for Mycoplasma contamination by PCR. Prior to each experiment, FCR3-CSA parasites were selected for CSA adhesion as previously described [13]. Ring stages of all strains were obtained by constant culture synchronization with 5% sorbitol and mature schizont-infected erythrocytes were purified by magnetic cell sorting LD columns (Miltenyi Biotec, Berg. Gladbach, Germany).

### Cytokine EASIA assay

In a 96 flat-bottomed well plate, NK92 cells (10^5^) previously cultured with recombinant human interleukin (rIL-)2 and without rIL-2 (24 hours starvation conditions) were co-incubated with 3D7 schizont-iRBCs (10 × 10^5^) and uninfected erythrocytes (10 × 10^5^) in RPMI-1640 medium (200 ul/well) at 37°C in 5% CO_2_. After 24 hours of incubation, IFN-γ was measured in the supernatants by a solid phase enzyme amplified sensitivity immunoassay kit (EASIA; Biosource). As controls, pure RPMI-1640 and supernatants of NK92 cells cultured in their normal growth medium (+ rIL-2) and under a 24 hours period of "starvation" (cell medium without rIL-2) as well as supernatants of iRBCs and uRBCs incubated without cells in RPMI-1640 were analysed for presence of IFN-γ. All samples were tested in duplicate according to the manufacturer's recommendations.

### NK92/iRBCs co-culture and flow cytometry

NK92 cells were kept in two different environments for 24 hours prior to the co-culture: in normal cell medium (+rIL2; NK92 nm) and in cell medium without rIL-2 (starvation medium; NK92s). Cells from both environments were co-cultured with 3D7 schizont-infected erythrocytes and uRBCs (NK92-RBCs ratio: 1:3) in their respective growth medium. As a positive control, cells were also incubated with a mixture of IL-12 and IL-18 (Peprotech and MBL, respectively; 100 ng/10^6 ^cells each). After the indicated time of incubation at 37°C and 5% CO_2_, NK cells from the co-culture as well as cells incubated without RBCs were stained for 30 min at 4°C with fluorochrome-conjugated antibodies for surface CD56 (APC), CD3 (PE), CD16 (FITC), CD69 (FITC), CD25 (PE) in parallel with the appropriate isotype controls. Cells were also internally stained with the IFN-γ (PE) antibody (all BD Biosciences). Dead cells were excluded from the analysis based on scatter signals and 7AAD fluorescence. Acquisition of samples was carried out in a FACS canto flow cytometer (BD Biosciences). Data were analysed with BD FACS Diva 6.0 software. Gates were set on the events compatible to lymphocytes regarding "size of the cells" × "internal complexity" (FSC × SSC). A total of 10.000 events were collected for each sample.

### Cytoadhesion assay

NK92 cells were incubated with 3D7 and FCR3-CSA schizont and ring-infected erythrocytes (NK - iRBCs ratio: 1:1, 1:3, 1:10) in RPMI 1640 medium, in a 6-well plate at 37°C and 5% CO_2 _for 1 h under continuous shaking. As a control for unspecific binding, the FCR3-CD36 strain was submitted to the same conditions. After incubation, the co-culture was stained with acridine orange and the adhesion of iRBCs to NK cells (rosettes) was observed by fluorescence microscopy.

### RNA isolation and microarray analysis of NK92 after co-culture

After 0, 6, 12 and 24 hours of co-culture of NK92 with 3D7-schizont-iRBCs or uninfected erythrocytes (uRBCs; 1:3) at 37°C in 5% CO_2_, RBCs were lysed (Lysis Buffer, BD) and NK cell RNA was isolated with RNeasy Mini Kit (Qiagen, Hilden, Germany). Quality of RNA specimen was validated on a Agilent BioAnalyzer 2100 (Agilent, Germany) and processed for Affymetrix Gene Chips using Affymetrix Whole Transcript Sense Target Labeling Kit (Affymetrix, Santa Clara, USA). Fragmented and labeled cDNA was hybridized onto human HuGene1.0 ST Gene Chips (Affymetrix, Santa Clara, USA). Staining of biotinylated cDNA and scanning of arrays were performed according to the manufacturer's recommendations. Analysis was done with 3 biological replicates. The MIAMI-compliant complete microarray data is deposited at the National Center for Biotechnology Information's (NCBI) Gene Expression Omnibus (GEO) under the entry name GSE 26876.

### Microarray data analysis

Raw data were imported into Expression Console 1.0 (Affymetrix, Santa Clara). Robust multichip average algorithm (RMA16, Bolstad 2003) was applied for array normalization and signal calculation [14]. Normalized signal values were imported into Genespring 11 (Agilent Technologies) and intensity values for the biological replicates were averaged for each time point and treatment. Significance was calculated using a Student *t *test without multiple testing correction, considering all transcripts with a minimum fold change in expression level of 1.5-fold together with a p-value < 0,05 compared to time point 0. Principal component analysis was performed based on the covariance matrix of normalized gene expression values to reduce the complexity of high-dimensional data structures and compare inter-variability of the array. Differentially expressed genes were annotated with Affymetrix database and their corresponding protein was ascribed. Lists of regulated genes were further analysed with Ingenuity Pathway Analysis (IPA). Expression profiles were visualized as heat maps using Genesis. Functional annotation of genes was performed according to the three gene ontologies (GO) describing gene products in terms of their "biological processes," "molecular functions" and "cellular compartments".

### Real-time PCR

Quantitative Real-time PCR was performed as described elsewhere on a Corbett Rotor-Gene Cycler (Corbett Research, New South Wales, Australia). The used primers were pre-designed Quantitect Primer Assays from Qiagen for the following genes of interest: CECR1, Fyb, KLRC2/C3, Lax, PTGDR and TNFSF4 (Ox40L). Experiments were ruled out in duplicate with samples from all three biological replicates. The specificity of primers was verified by melting curve analyses and all had similar amplification efficiencies. mRNA levels were normalized to glycerinaldehyde-3-phosphate dehydrogenase (GAPDH), and expressed relative to control samples at time point 0 using the 2^-ΔΔCT ^method.

### Growth inhibition assay

3D7 or Dd2 sorbitol-synchronized ring-stage-iRBCs were co-incubated with NK92 (pre-cultured overnight without IL2) in different ratios in parasite growth medium, 0,125% starting parasitaemia and 1% haematocrit. As controls, co-culture was also performed with HeLa cells, Jurkat cells and C32 cells under the same conditions. After 24 to 48 hours of incubation at 37°C in parasite atmosphere, culture samples were frozen at -20°C, then thawed and parasite growth inhibition was quantified by a Histidine-Rich Protein 2 (HRP2) ELISA assay performed as described elsewhere [15].

### Statistical analysis

Analysis was performed using StatView for Windows 5.0.1 (SAS Institute Inc., Cary, North Carolina) running on Windows XP (Microsoft Corp., Redmond, Washington). Results were analysed using either ANOVA test (*p *< 0,05) or Bonferroni test (*p <*0,005).

## Results

### NK92 cells constitutively release IFN-γ

**Figure 1:**
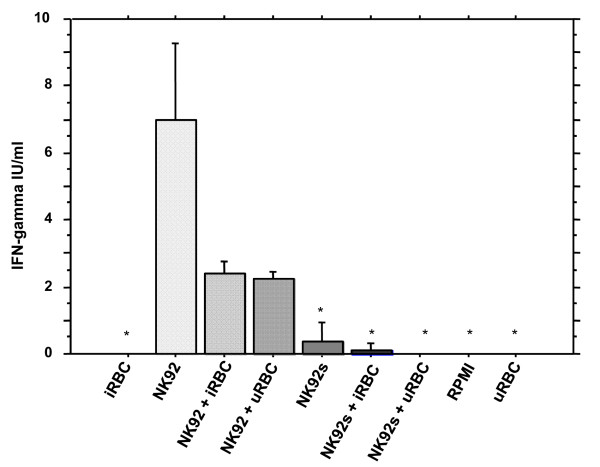
**IFN-γ release of NK92 cells after co-culture with iRBCs**. NK92 cells previously cultured with rIL-2 and without rIL-2 (24 hours starvation; NK92s) were co-incubated with 3D7 schizont-iRBCs and uninfected erythrocytes in RPMI-1640 medium (24 hours - 37°C - 5%CO_2_) and supernatants were subsequently tested for IFN-γ release. iRBCs/uRBCs: supernatants from iRBCs and uRBCs incubated without cells in RPMI-1640; NK92/NK92s: supernatant from cells incubated in normal growth medium and starvation medium, respectively. Values represent mean ± S.E.M.; *p = 0.0001 for comparison with NK92 (*p <*0,05 - ANOVA).

To assess whether NK92 cells release IFN-γ upon contact with iRBCs, *in vitro *24 hours co-cultures of NK92 with 3D7-iRBCs were performed and supernatants were analysed for cytokine presence (Figure 1). NK92 cells grown alone in normal cell medium released 7 IU/ml of IFN-γ compared to 2,4 IU/ml and 2,3 IU/ml when iRBCs and uninfected erythrocytes (uRBCs), respectively were added to the assay. Only a very low level (0,2 IU/ml) of IFN-γ was detected in the supernatant of NK92 cells that were maintained under starvation conditions (NK92s). The addition of iRBCs and uRBCs to NK92s decreased the amount of IFN-γ released. As controls, supernatants of iRBCs, uRBCs and RPMI 1640 were also tested and did not present traces of IFN-γ. Significant differences were seen when the levels of IFN-γ released by the cells cultured in normal medium without stimulus was compared to that released by iRBCs alone, NK92s, NK92s + iRBCs, NK92s + uRBCs and uRBCs (*p *< 0,0001). To sum up, NK92 cells that were submitted to starvation before the experiment did not produce IFN-γ due to co-culture with iRBCs. In addition, cells kept in normal culture conditions (+rIL2) prior to the experiment released greater levels of IFN-γ regardless presence of iRBCs.

### 3D7 parasites do not induce up-regulation of activation markers in NK92 cells

To further investigate NK92 expression of activation markers due to iRBCs contact, *in vitro *co-cultures were carried out for 24 hours and cells were analysed by flow cytometry. Independently of the medium that NK92 cells were incubated (normal cell medium or starvation medium) the NK phenotypic marker CD56 was always positive, CD3 and CD16 surface antigens were always negative, proving that the cells presented the NK phenotype (Table 1). The activation markers CD25 and CD69 were already positive without addition of stimulus, indicating certain "base level" activation. However, the addition of IL12+IL18 (positive control) induced up-regulation of both activation markers as well as IFN-γ above the "base level" activation, showing that these cells are able to respond to an external stimulus.

**Table 1:** FACS analysis of the NK92 cell line after co-culture with 3D7

Starvation	NS (%/MFI/SD)	uRBCs (%/MFI/SD)	iRBCs (%/MFI/SD)	IL (%/MFI/SD)
CD56	100/51177/26893	100/47072/22400	100/48515/23025	100/62661/30093
CD3	0.1/142/105	0.1/133/103	0.1/135/97	0.7/182/125
CD16	0.5/599/347	0.5/601/389	0.5/605/373	10/724/405
CD25	94.2/3834/2269	95.6/3570/1983	96.5/3809/2040	98.3/14259/12340
CD69	3.4/824/635	1.7/747/473	2.7/800/568	58.1/3883/3127
IFN-γ	0.1/202/102	0.1/189/98	0.1/192/98	40.4/2270/4658

**Normal Medium**	**NS (%/MFI/SD)**	**uRBCs (%/MFI/SD)**	**iRBCs (%/MFI/SD)**	**IL (%/MFI/SD)**

CD56	99.9/79722/37294	100/72957/35805	100/78647/65793	100/68544/34844
CD3	1.4/209/135	1.7/235/146	1.3/215/137	7.5/302/232
CD16	2.3/859/473	3.2/963/548	2.8/929/544	8.5/1195/792
CD25	6.2/313/722	5.2/318/762	5.6/309/584	95.9/188865/19512
CD69	39.7/2218/1804	44.5/2319/1655	48.5/2485/1904	84.1/5950/4900
IFN-γ	1.6/297/152	1.1/270/130	1.2/291/201	75.3/3504/4701

NS: no stimulus; uRBCs: uninfected erythrocytes; iRBCs: infected erythrocytes; IL: positive control; %: percentage of positive cells; MFI: mean fluorescence intensity; SD: standard deviation

As expected, co-culture of NK92 with uRBCs did not change the picture described above. No up-regulation of CD25, CD69 and IFN-γ was observed in comparison to the cells cultured without RBCs. Surprisingly, the addition of 3D7-infected erythrocytes to the system also did not have any significant impact on NK cells. No up-regulation of CD25 and CD69 was detected due to the addition of iRBCs although these membrane proteins, especially CD69, should be the first markers expressed in immune activated cells. In addition, no significant up-regulation of IFN-γ was observed (Table 1).

### NK92 form rosettes with FCR3-CSA-iRBCs but not with 3D7

**Figure 2:**
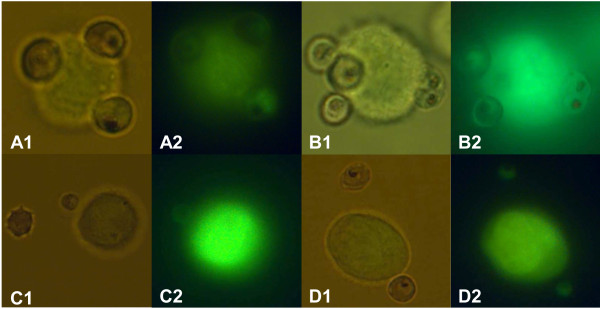
**Rosettes of NK92 and FCR3-CSA parasites**. NK92 were co-cultured with FCR3-CSA-iRBCs in 1:3 ratio respectively, for 1 h at 37°C/5%CO_2_. After incubation, a sample of the co-culture was dyed with acridine orange and analysed in a fluorescence microscope. **A1 and B1**. Rosettes of 1 NK cell with 2-3 iRBCs (normal light). **A2 and B2**. The same rosettes in fluorescent light. **C1, C2, D1, D2**. NK92 and FCR3-CD36 as a control for unspecific binding. The binding is specific for infected erythrocytes. Few uninfected RBCs were found to bind the NK cells.

Baratin *et al *described rosettes as conjugates of NK cells with more than two RBCs and that this direct contact with iRBCs could contribute to complete activation of NK cells [16]. In this study, formation of rosettes between 3D7 and selected FCR3-CSA with NK92 in different ratios (1:1, 1:3 and 1:10; NK:RBC) was tested. For the 3D7 strain, no rosettes could be observed in none of the tested ratios. Best results were obtained with FCR3-CSA in 1:10 ratio. Ten percent of NK92 formed conjugates with iRBCs (Figure 2A and 2B). Nevertheless, these conjugates were mostly not real rosettes since mainly one RBC per NK cell was bound. Only around 1% of the cells were able to form rosettes with more than two attached iRBCs. There was no binding of NK92 with uRBCs and no rosettes were formed with FCR3-CD36 (Figure 2C and 2D).

### *Plasmodium falciparum-*iRBCs influence NK92 gene expression only after 24 hours, but not at 6 or 12 hours of co-culture

**Figure 3:**
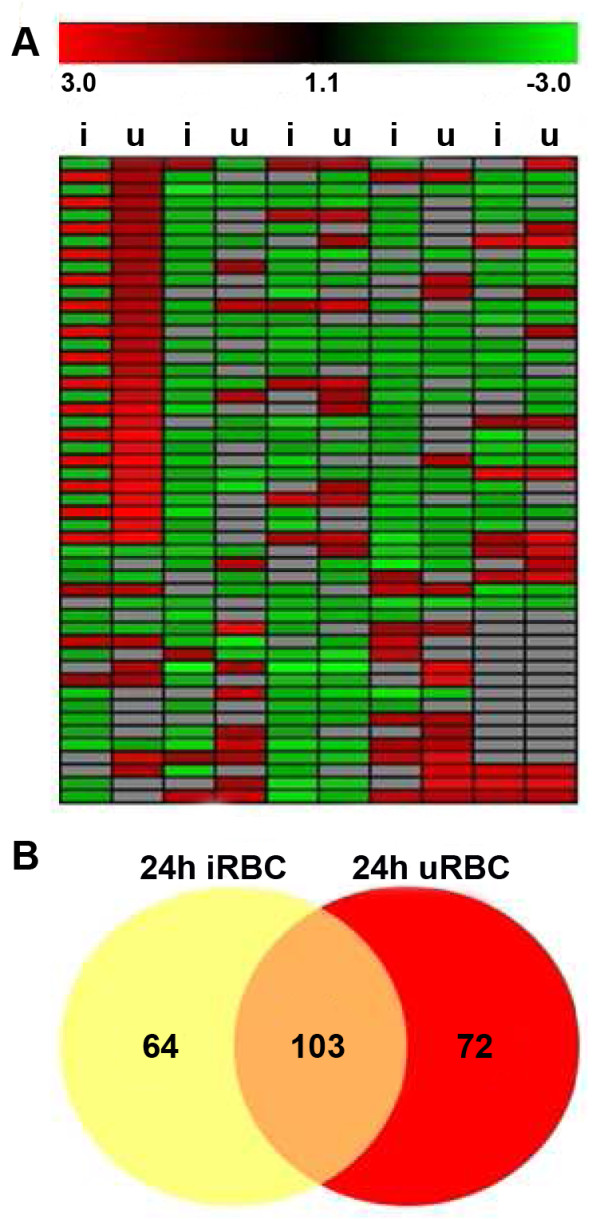
**Gene expression patterns in NK92 after 24 h co-culture with *P. falciparum-*iRBCs**. Normalised expression patterns were derived from microarrays of NK92 cells after 24 hours co-culture with 3D7-iRBCs or uRBCs (1:3). Data were obtained from 3 biological replicates **A**. Heatmap of altered genes in 24 h co-cultures normalised to untreated NK92 at time point 0 h comparing uRBCs (u) and iRBCs (i)-treated NK92. Up-regulated genes are depicted in red and down-regulated in green. **B**. Venn-diagram of total numbers of the same genes. Only regulated genes with a fold-change ≥ 1.5 (p ≤ 0,05) are depicted. 167 genes were differentially expressed in the NK92 cells after co-culture with iRBCs, of which 85 genes were up-regulated and 82 down-regulated. However, there is a big overlap of 103 genes with NK92 cells co-cultured with uRBCs.

**Figure 4:**
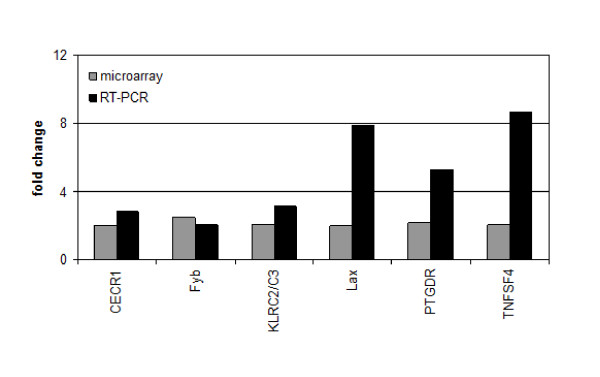
**RT-PCR validation of microarray results**. Fold change in gene expression of NK92 cells after 24 hours of 3D7-iRBCs co-culture comparing microarray and qRT-PCR results for 6 genes of interest (CECR1, Fyn-binding protein, KLRC2/C3, LAX, PTGDR, and TNFSF4 (Ox40L)) linked to priming of adaptive immune responses, NK granule secretion and cell activation.

To avoid missing the right time point of transcriptional changes induced by *P. falciparum-*iRBCs, we performed a time kinetics experiment investigating transcription in NK92 cells after 6, 12 and 24 hours of co-culture with parasites. Expression data show almost no change on transcription level after 6 h (10 genes) and 12 h (12 genes) of co-cultivation compared to control (time point 0). After 24 h of co-culture a total of 167 genes were differentially expressed in the NK92 cells (Figure 3). However, there is a big overlap of 103 genes with NK92 cells co-cultured with uRBCs. Since we cannot use 100% iRBCs for experiments, this overlap can be explained by the influence of the uRBCs present in the iRBCs co-cultures. Only 64 genes were regulated due to iRBCs. Out of these 64 genes, 53 were up-regulated (24 mitochondrial genes) and 11 down-regulated. Analysis of these genes with Ingenuity revealed "cancer/respiratory disease", "cell-to-cell signalling" and "interaction/cell-mediated immune response" as well as "cell cycle/infection mechanism and inflammatory response" as top networks with a score ≥ 25 (Table 2). The majority of up-regulated genes are linked to the biological process of cell-cycle progression and possible entry into G2-phase, an event characterized by cell growth as well as protein and RNA biosynthesis (Table 3). Many guide-RNAs required for splicing were up-regulated (SCARNA7 and 9, SNORA40/JOSD3, SNORD47/GAS5, SNORD50B and SNORD75). Furthermore, genes involved in anti-apoptosis and cell growth (GIMAP5, FAIM3, ZNF780A, Tubulin-y, MT1E and CECR1) were over-expressed. Another set of interesting genes is linked to immune response and activation of NK cells, especially granule secretion (CECR1, TNFSF4, KLRC2/C3, Centaurin delta 1, Fyb, PTGDR, Tubulin-β). Microarray results were validated by RT-PCR for some representative genes (CECR1, Fyb, KLRC2/C3, LAX, PTGDR and TNFSF4). Normally the fold change was always higher for the RT-PCR analysis (Figure 4).

**Table 2:** Top networks related to *P.falciparum*-induced NK92 genes

*Associated Network Functions*	*Score*
Cancer, Genetic Disorder, Respiratory Disease	49
Cell-Cell Signaling/Interaction, Cell-Mediated Immune Response, Cellular Development	28
Cell Cycle, Cellular Compromise, Infection Mechanism	25
Cell Cycle, Nervous System Development and Functions, Inflammatory Response	25
Cell Cycle, Cellular Assembly/Organization, DNA Replication, Recombination and Repair	24

**Table 3:** Top bio-functions related to *P.falciparum -*induced NK92 genes

*Molecular and Cellular Functions*	*p-Value*	*N° Molecules*
Cell Cycle	3,41E-09 - 3,51E-02	34
DNA Replication, Recombination and Repair	2,31E-06 - 3,00E-02	36
Cellular Assembly and Organization	5,27E-06 - 3,18E-02	21
Gene Expression	5,69E-05 - 3,00E-02	13
Cell Death	6,58E-05 - 3,00E-02	32

***Physiological System Development and Function***	***p-Value***	***N° Molecules***

Embryonic Development	2,30E-05 - 3,19E-02	7
Connective Tissue Development and Function	2,30E-05 - 3,19E-02	11
Cell-mediated Immune Response	1,16E-03 - 1,83E-02	10
Hematological System Development and Function	1,16E-03 - 3,00E-02	18
Hematopoiesis	1,16E-03 - 2,80E-02	12

### Dd2 and 3D7 parasitaemia decreases significantly after parasite incubation with NK cells and Jurkat cells

**Figure 5:**
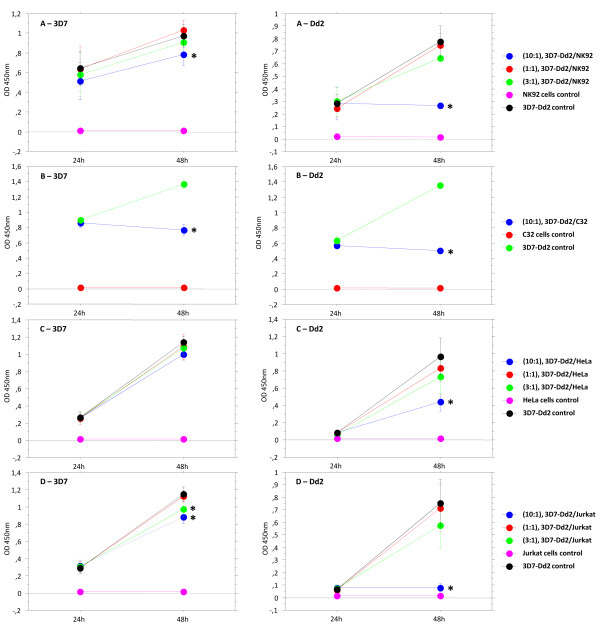
**Parasite growth inhibition assay**. 3D7 (left column) or Dd2 (right column) sorbitol-synchronized ring-stage-iRBCs were co-incubated with NK92 (A) in different ratios (10:1, 1:1, 3:1 - parasites:cells) in parasite growth medium. Starting parasitaemia was 0,125% in 1% haematocrit. As controls, co-culture was also performed with C32 cells (B), HeLa cells (C), and Jurkat cells (D) in the same conditions. After 24 hours to 48 hours of incubation at 37°C in parasite atmosphere, parasite growth inhibition was quantified by measuring HRP2 release by ELISA. *p < 0.002 for paired comparisons (*p < 0.005 *- Bonferroni).

In order to investigate whether NK cells affect parasite growth, invasion and/or development, *in vitro *co-cultures were carried out with different NK - iRBCs ratios. Parasite growth was significantly inhibited due to co-culture with NK cells after 48 h especially at the 10:1 ratio (3D7/Dd2:NK92). The Dd2 growth was suppressed in a greater extent (*p <*0,0001) than the 3D7 growth at such ratio (*p *= 0,0018) in comparison to the controls. Development of both strains was slightly but not significantly inhibited at 3:1 ratio while at 1:1 ratio no differences were observed in comparison to the controls (Figure 5A).

To verify whether the observed growth inhibition was due to a specific response of NK cells, we have performed the same co-culture experiment with diverse cell lines. C32 cells have equally suppressed growth of 3D7 and Dd2 (*p <*0,0001) after 48 h co-culture in comparison to controls (Figure 5B). In contrast, HeLa cells restrained Dd2 development to a larger extent at 10:1 ratio (Dd2:HeLa; *p <*0,0001) although no significant effect of such cells was observed on 3D7 growth (Figure 5C). In the presence of Jurkat cells, Dd2 parasitaemia was drastically decreased (*p <*0,0001) at the 10:1 ratio (Dd2:Jurkat) in comparison to the control. In addition, the growth curve of the 3D7 parasites was considerably inhibited in comparison to the control at 10:1 (3D7:Jurkat; *p <*0,0001) and 3:1 (*p <*0,0002) co-culture ratios (Figure 5D).

**Figure 6:**
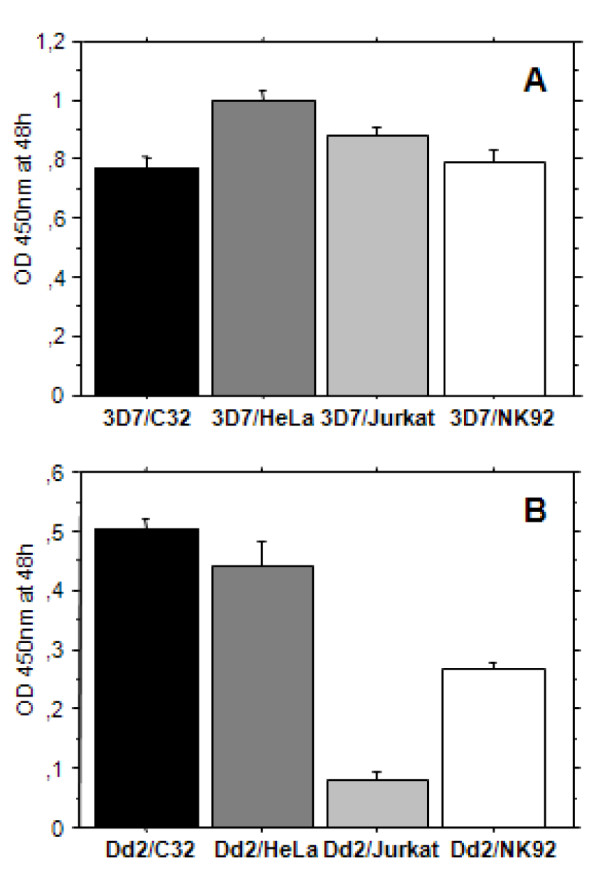
**3D7 and Dd2 growth inhibition induced by different cell lines**. 3D7 or Dd2 sorbitol-synchronized ring-stage-iRBCs were co-incubated with NK92, C32 cells, HeLa cells and Jurkat cells in the same conditions. After 24 to 48 hours of incubation, parasite growth inhibition was quantified by measuring HRP2 release by ELISA. Comparison of the inhibition effect of each cell line on 3D7 **(A) **and Dd2 **(B) **is depicted.

Comparing the effects that all four cells lines imposed on parasitaemia after 48 hours, Dd2 was observed to be more sensitive to the co-cultures than 3D7 (Figure 6). NK92 and Jurkat cells showed a stronger impact than C32 and HeLa cells on Dd2 growth. Moreover, NK92 and C32 cells have similarly affected 3D7 development in a stronger manner than HeLa and Jurkat cells. Taken together, these data shows that all cell lines influenced parasite growth at 10:1 (parasite:cell) ratio except for the HeLa:3D7 co-culture; that Dd2 is affected by the cells in a greater extent than 3D7 and that the impact observed on *in vitro *parasite growth is therefore not NK cell-specific.

## Discussion

PBMCs are frequently adopted tools for studies of immune responses in malaria. NK cell cross talk with accessory cells triggers their activation and effector functions. In this study, however, a reductionist approach (without accessory cells) was chosen, aiming to investigate the very specific effect of NK92 cells towards *Plasmodium *parasites.

NK92 cells were already producing IFN-γ when incubated alone in normal growth medium. This is probably due to the presence of IL-2, a cytokine already reported to induce IFN-γ secretion in mice macrophages [17]. In addition, external and intrinsic factors might also play a role in the release of IFN-γ. The handling of the cultures and the fact that these are tumour/continuous cells (and for this reason already went through a process of activation) has to be taken into account. Co-incubation with iRBCs did not induce IFN-γ release by NK92 cells as a response to *P. falciparum *antigens. It was already shown that signals from accessory cells such as macrophages, monocytes and dendritic cells (DCs) are required for full NK cell commitment. Moreover, it is known that PBMCs from malaria-unexposed donors can produce heterogeneous responses, including IFN-γ release, when stimulated by iRBCs [4-6]. A possible explanation to these results is, of course, the lack of accessory cells but it might be possible that the donor of such NK cells is a low IFN-γ responder [5]. If this is the case, accessory cells in the system would not change the picture. In a study using NK92 cell lines as models for IL-18-mediated signal transduction it was shown by RT-PCR and ELISA that the activation of the cell lines with IL-18 alone failed to stimulate IFN-γ protein production despite inducing expression of IFN-γ mRNA [18]. By the NK92 microarrays however, no induction of the IFN-γ gene up to 24 hours of co-culture with parasites was observed. It would be interesting, therefore, to test whether mRNA expression can be detected at a later time point.

CD69 is a C-type lectin-like glycoprotein known to be a sensitive early marker of leukocyte activation and cytotoxic activity of NK cells [19]. However, only a slight up-regulation of CD25, another activation marker, was observed when NK92 grown under starvation conditions were incubated with iRBCs. The same was detected when uRBCs were added to the system suggesting that the weak activation detected is not iRBCs-specific. Genetic differences between people appear to influence NK cell response to iRBCs. It is claimed that relevant gene(s) may be variably expressed among different NK clones [5] which might, therefore, influence NK cell activation. In addition, no binding of NK92 with 3D7-iRBCs was detected although physical interactions between NK cells and iRBCs have already been described with freshly isolated NK cells and NK cell lines [4,5,16,20]. However, real rosettes were observed with FCR3-CSA-iRBCs what might explain a probable engagement of *var2csa *on the surface of iRBCs. It was suggested that CSA, claimed to be involved in pregnancy-associated malaria [21] is the element through which PfEMP1 of FCR3-CSA strain will form the rosettes. If this is the only factor involved in that event, it explains the reason that adherence with 3D7 was not detected.

The microarray results suggest that NK92 cells proliferate in response to *P. falciparum-*iRBCs after 24 hours of co-culture. Another study on mice experimental malaria already reports a similar observation [22]. After an early interferon type-I response a second wave of differential expression was apparent at 24-32 hours post infection with *Plasmodium chabaudi*. Such expression was linked to NK cell proliferation in the peripheral blood although signs of activation were absent. Interestingly, the same pattern was observed in the present study at 24 hours, since an activated status of NK92 cells after iRBCs co-culture could be detected neither by microarray analysis nor by flow cytometry. Of course, not the same set of genes was found at earlier time points, because in this study no accessory cells or any additional cytokines were applied. However like in another report, genes important for DNA replication, cell proliferation, spindle formation and microtubule cytoskeleton formation were altered. In the study of Kim *et al*. the main KEGG pathways were cell cycle and small cell lung cancer comparable to the pathways of cancer/respiratory disease and cell cycle (infection mechanism/inflammation) presented in this study [22].

In addition to the proliferative signs, few other altered genes gave a hint for possible NK cell triggering. After 24 hours KLRC2 and KLC3 were up-regulated, both activating NK cell receptors. KLRC2 together with CD94 is involved in NK cell-mediated cytotoxicity and ligand binding leads to granule release as well as TNF-α and IFN-y secretion. Up-regulation of CECR1 in NK92 cells might be a sign for T cell priming since it was shown that inhibition will lead to less signal transduction via CD3 and TCR [23]. Another important gene in this context is TNFSF4 (OX40L): it serves as a ligand for OX40 and results in higher CD4^+ ^T cell proliferation and cytokine production, especially IFN-y. It is selectively induced in IL-2/-12 or -15 treated NK cells after stimulation via NKG2D, CD16 or KIR2DS2 [24]. Additionally, Centaurin delta 1 that signals via phosphatidylinositol-3-kinase pathway to induce cytoskeleton remodelling and thus influencing granule secretion was altered [25]. Also interesting in this context is FYB, a Fyn-binding protein, which can phosphorylate IKKa/b and ubiquitinylate IKKy resulting in NF-kB activation in T cells [26] and degranulation. IKK was one of the few up-regulated genes after 6 hours. Among the few down-modulated genes is tubulin-β. Tarazona *et al *have reported an important role in killing of target cells, cell polarisation, cellular movement and granule secretion [27]. Furthermore, PTGDR expression was up-regulated. This receptor is known to increase intracellular cAMP concentration and subsequently inhibit NK cell function through blocking Th1-cytokine production and cytotoxicity/promotion of Th2-type responses [28]. Although not many conclusions can be drawn from these results, it is possible that the adaptive immunity is primed and NK cells become activated by iRBCs to release their granule contents. However, complete pathways linked to activation were not found to be switched on in the present study. Still, the microarrays showed the existence of similar mechanisms altered in human NK cells after *P. falciparum*-iRBCs encounter comparable to those reported from mouse model experiments.

To validate the picture observed with the arrays, the impact of NK92 cells on parasite growth, invasion and development upon co-culture was investigated. The experiments show that NK92 cells interfere with the parasite life cycle, especially with that from the Dd2 strain. In addition, Jurkat cells, a T cell line, strongly diminished Dd2 parasitaemia. These results would be in concert with the general knowledge that immune responses against malaria parasites relies upon NK and T cells [1,2]. Surprisingly, however, other cell types irrelevant to immune response in malaria (C32 and HeLa) also suppressed parasite development. These results might suggest that the inhibition of parasite growth caused by NK92 cells is an effect of competition for limited resources in the presence of growing and dividing cells. Upon co-culture however, cells were kept at usual and viable concentrations for this type of assays, as commonly performed in experiments were fresh cells are used. It could be observed that the decrease of parasitaemia caused by irrelevant cells was somehow related to the parasite strain used. HeLa cells for example had a very strong impact on Dd2 parasitaemia but did not influence growth of 3D7 parasites. The same is true for the C32 cells. NK92 and Jurkat cells had an even stronger impact on the growth of Dd2. However, due to the lack of a cell-specific response, these results cannot be claimed to reflect a cytotoxic effect of NK92 cells against parasites. However, if the effect is there, it appears to be subtle and is not comparable to that imposed by primary NK cells.

If characteristics from primary NK cells could be extrapolated to NK cell lines, one could assume that NK92 cells belong to the CD56bright sub-population, which is known to be CD16dim/neg. These cells cannot elicit ADCC but are potent IFN-γ producers what is then in accordance to the results presented in this study (cells release IFN-y even without stimulus). The lack of parasite-induced activation could be solely linked to the fact that no accessory cells were adopted. In addition, a more specific NK cell effect against parasites might have been detected if NK92 cells belonged to the CD56dim population, which is known to be important for their cytotoxicity against *Plasmodium*.

## Conclusions

On the one hand, this study shows that there is a lack of primary signs of NK92 activation in response to *Plasmodium *stimulus although NK92 transcription of proliferation- and priming-related genes was clearly changed in response to such interaction. On the other hand, a drastic impact on *P. falciparum *parasitaemia linked to NK cell contact was observed. Whether it reflects only consequences of competition with the co-cultured cells or whether there is the addition of the cytotoxic effects of NK92 needs to be further investigated. Although NK92 alone were observed to disqualify as good models for the NK immune response to *Plasmodium*, interesting information regarding the mechanisms behind NK effector responses to parasites were acquired and will be of use for future basic research in malaria.

## Competing interests

The authors declare that they have no competing interests.

## Authors' contributions

EGC and EB designed the study, carried out the laboratory work, analysed and interpreted the data, wrote the manuscript; VTH carried out the laboratory work and analysed the data; PGK provided scientific leadership and corrected the manuscript; JFJK provided scientific leadership, designed the study, analysed the data, wrote the manuscript. All authors read and approved the final manuscript.
